# *Torque Teno Sus Virus* (TTSuV) Prevalence in Wild Fauna of Northern Italy

**DOI:** 10.3390/microorganisms10020242

**Published:** 2022-01-22

**Authors:** Francesco Righi, Sara Arnaboldi, Virginia Filipello, Giovanni Ianiro, Ilaria Di Bartolo, Stefania Calò, Silvia Bellini, Tiziana Trogu, Davide Lelli, Alessandro Bianchi, Silvia Bonardi, Enrico Pavoni, Barbara Bertasi, Antonio Lavazza

**Affiliations:** 1 Istituto Zooprofilattico Sperimentale della Lombardia e dell’Emilia Romagna (IZSLER), 25124 Brescia, Italy; francesco.righi@izsler.it (F.R.); virginia.filipello@gmail.com (V.F.); stefania.calo@izsler.it (S.C.); silvia.bellini@izsler.it (S.B.); tiziana.trogu@izsler.it (T.T.); davide.lelli@izsler.it (D.L.); enrico.pavoni@izsler.it (E.P.); barbara.bertasi@izsler.it (B.B.); antonio.lavazza@izsler.it (A.L.); 2 National Reference Centre for Emerging Risks in Food Safety (CRESA), Istituto Zooprofilattico Sperimentale della Lombardia e dell’Emilia Romagna (IZSLER), 20133 Milan, Italy; 3 Emerging Zoonoses Unit, Department of Food Safety, Nutrition and Veterinary Public Health, Istituto Superiore di Sanità, 00161 Rome, Italy; giovanni.ianiro@iss.it (G.I.); ilaria.dibartolo@iss.it (I.D.B.); 4 Istituto Zooprofilattico della Lombardia e dell’Emilia Romagna (IZSLER), 23100 Sondrio, Italy; alessandro.bianchi@izsler.it; 5 Veterinary Science Department, Università degli Studi di Parma, 43100 Parma, Italy; silvia.bonardi@unipr.it

**Keywords:** swine-related virus, wild ungulates, reservoir

## Abstract

*Torque teno sus virus* (TTSuV) is a non-enveloped circular ssDNA virus which frequently infects swine and has been associated with hepatic, respiratory, and autoimmune disorders. TTSuV’s pathogenic role is still uncertain, and clear data in the literature on virus reservoirs are lacking. The aims of this study were to investigate the presence of potentially zoonotic TTSuV in wild animals in Northern Italy and to evaluate their role as reservoirs. Liver samples were collected between 2016 and 2020 during four hunting seasons from wild boars (*Sus scrofa*), red deer (*Cervus elaphus*), roe deer (*Capreolus capreolus*), and chamois (*Rupicapra rupicapra*). Samples originated from areas in Northern Italy characterized by different traits, i.e., mountains and flatland with, respectively low and high farm density and anthropization. Viral identification was carried out by end-point PCR with specific primers for TTSuV1a and TTSuVk2a species. TTSuV prevalence in wild boars was higher in the mountains than in the flatland (prevalence of 6.2% and 2.3%, respectively). In wild ruminants only TTSuVk2a was detected (with a prevalence of 9.4%). Our findings shed light on the occurrence and distribution of TTSuV in some wild animal species, investigating their possible role as reservoirs.

## 1. Introduction

*Torque teno viruses* (TTVs) belong to the *Anelloviridae* family and are widespread worldwide in humans and animals [1,2]. TTVs were isolated for the first time in humans in 1997 in a patient with post-transfusional hepatitis of unknown etiology [3]. Human infection has been associated with respiratory diseases, acute enteritis, viral hepatitis, and autoimmune rheumatic diseases [4,5,6,7]. Their pathological mechanisms are still unclear, but recent studies suggested an interaction with the host immune system. Moreover, in the case of co-infection with other viruses an increased disease severity was reported [8].

TTVs are ubiquitous viruses and have been often detected in mammalian species, including dogs, cats, swine, cattle, sheep, wild boars, hares, and non-human primates [9,10,11,12,13,14]. TTVs are genetically distinct and are classified in a species-specific manner, although the genomes of TTVs detected from several animal species, including humans, show a similar organization [15]. Since a human cross-species infection of TTVs of animal origin cannot be excluded, it seems interesting to investigate the possible role of wild animal species as reservoirs.

In swine, *Torque teno sus virus* (TTSuV) has a genome organization similar to human TTV (huTTV) and comprises two genera: *Iotatorquevirus*, which includes TTSuV1a, and *Kappatorquevirus*, which includes two species (TTSuVk2a and TTSuVk2b [2,16,17,18,19]. TTSuV1a and TTSuVk2a are the most studied and well-characterized genogroups and appear to act as primary pathogens in swine, causing mild to moderate respiratory, hepatic, and nephritic lesions [15,20,21]. Recently, TTSuV co-infection with other swine-related viruses was studied, and it was demonstrated that the association of TTSuV with Porcine circovirus type 2 can enhance disease severity [22].

TTSuVs are mainly transmitted by the fecal–route, and they are frequently detected in fecal excretions as well as nasal excretions, sera, and several organs including the liver of infected pigs [23]. The virus transmission may also occur by a vertical route, as fetuses infected with TTSuV have been found at different stages of pregnancy [24,25].

TTSuV is widespread in Europe in both swine and wild boars, as reported by different studies: TTSuV was found in Romania and in Germany, while a high sero-prevalence (84%) was found among wild boars in Spain [12,26,27]. In Italy, previous studies reported a prevalence of 83.2% in sera of pigs at different ages, as well as a prevalence of 58.3% in fresh pork liver sausages [28,29,30]. However, studies investigating the role of wild animals, other than wild boars, as TTSuV reservoirs, are lacking.

The aims of this study were to investigate the occurrence of potentially zoonotic TTSuV1a and TTSuVk2a in wild ungulates in Northern Italy and to evaluate their potential role as reservoirs.

## 2. Materials and Methods

### 2.1. Sampling

Liver samples were collected during the hunting seasons between 2016 and 2020 from wild ungulates, including wild boars (*Sus scrofa*), red deer (*Cervus elaphus*), roe deer (*Capreolus capreolus*), and chamois (*Rupicapra rupicapra*). 

Two areas in Northern Italy with different geographical and environmental features (Sondrio and Parma Provinces) were investigated. Sondrio Province is characterized by mountains, low anthropization of the territory, low farm density, and high conservation of the ecosystem. Indeed, in this area wild boar is allochthonous, and therefore subjected to eradication, since its presence is due to illegal repopulation for poaching. In this area, other wild ruminants are also present at quite high densities: monitoring studies reported about 15 deer/100 ha, 5–10 roe deer/100 ha, and 3–10 chamois/100 ha [31,32]. The other sampling area near Parma in the Po Valley was a highly anthropic area, with a high pig farm density. In this area wild boars are widespread (about 12 animals/100 ha), and depopulation interventions are routinely planned [33]. Red deer, roe deer, and chamois livers were not collected from this area since their presence is sporadically or never (chamois) reported.

A total of 528 liver samples were collected from wild ungulates of both genders and divided into three age classes: class 0—young animals (less than 1 year-old), class 1—sub-adults (between 1 and 2 years of age), and class 2—adults (more than 2 years-old). A total of 400 samples were collected from wild boars in the two areas between the 2018 and 2020 (*n* = 169 in 2018–2019 hunting season and *n* = 231 in the 2019–2020 hunting season, Table 1). The other 128 liver samples were collected from red deer, roe deer, and chamois in the Sondrio Province between the 2016 and 2019 hunting seasons (*n* = 58 in 2016–2017, *n* = 32 in 2017–2018 and *n* = 38 in 2018–2019, Table 2). 

### 2.2. Sample Preparation and DNA Extraction

For TTSuV detection, 50 g of hepatic tissue was accurately minced in a laboratory blender, and a 25 mg aliquot was weighed for the genome extraction. Viral DNA was extracted using the NucleoSpin^®^ Tissue kit (Marchery-Nagel, Düren, Germany), according to the manufacturer’s instructions. The eluted DNA (100 µL) was stored at −80 °C until use.

### 2.3. Typing PCR

TTSuV1a and TTSuVk2a were detected by two end-point PCRs using specific primers targeting the TTSuV’s highly conserved UTR region [34] (Table 3).

The reaction was performed for both PCRs in a total volume of 18 µL containing 3 µL of 1X Green GoTaq Flexi Buffer (Promega, Madison, WI, USA), 1 µL of MgCl2 (2 mM), 0.3 µL of dNTPs pool (0.4 mM), 0.6 µL of primers (0.2 µM each), 0.1 µL of Go Taq Flexi DNA polymerase (0.025 U/µL, Promega, Madison, WI, USA), 8 µL of DNAse-RNase-free water (Sigma-Aldrich, St. Louis, MO, USA); and finally, 5 µL of extracted DNA was added to each reaction mix. Negative and positive controls were included in each run.

PCRs were performed on a GeneAmp^®^ PCR System 9700 thermal cycler (Applied Biosystems Inc., Foster City, CA, USA) as follows: 2 min at 95 °C, 40 cycles of 30 s at 95 °C, 30 s at 54 °C and 15 s at 72 °C, and a final incubation at 72 °C for 5 min.

PCR products and 100 bp ladder (Invitrogen, Carlsbad, CA, USA) were loaded into a 2.5% agarose gel (Roche, Basel, Switzerland), stained with EuroSafe Nucleic Acid Stain (EuroClone SpA, Milan, Italy). The results were observed through a FireReader V10 transilluminator (UVITEC, Cambridge, UK).

### 2.4. Sequencing

The positive samples were sequenced to confirm the virus identification. The amplification products were first enzymatically purified using FastAP™ Thermosensitive Alkaline Phosphatase (1 U/µL) and Exonuclease I (20 U/µL) (Thermo Fisher Scientific, Waltham, MA, USA), according to the manufacturer’s instructions.

The forward and reverse sequence reactions were prepared separately using the primers listed in Table 3 on a GeneAmp^®^ PCR System 9700 thermal cycler (Applied Biosystems Inc., Foster City, CA, USA). Each reaction was performed in a total volume of 10 µL containing 2 µL of Big Dye Terminator Reaction Mix (Thermo Fisher Scientific, Waltham, MA, USA), 1 µL of Big Dye Terminator Sequencing Buffer (Thermo Fisher Scientific, Waltham, MA, USA), 3 µL of DNAse-RNase-free water (Sigma-Aldrich, St. Louis, MO, USA), 2 µL of primer (1.6 µM), and 2 µL of purified product. Samples were incubated at 96 °C for 1 min and amplified for 25 cycles of 96 °C for 10 s, 50 °C for 5 s, and 60 °C for 4 min.

Sequence reaction products were purified using the BigDye XTerminator^®^ Purification Kit (Thermo Fisher Scientific, Waltham, MA, USA), according to the manufacturer’s instructions. Samples were then sequenced on a SeqStudio Genetic Analyzer (Applied Biosystem, Foster City, CA, USA).

The consensus sequences were created and aligned with MEGAX software [35,36]. The generated sequences were compared for similarity versus all virus sequences in the NCBI GenBank database using the Basic Local Alignment Search Tool (BLAST).

### 2.5. Statistical Analysis

To calculate the prevalence of and to identify the risk factors for the virus, a sample was considered positive when at least one of the two virus species was detected (TTSuV1a or TTSuVk2a). The apparent TTSuV prevalence was calculated with the corresponding 95% confidence intervals (95% CI) estimated using an exact method. 

To investigate the effects of the risk factors on TTSuV, the chi-squared test was used. In particular, the relationship is statistically significant if the *p*-value is less than 0.05 (*p*-value < 0.05).

All statistical analysis were carried out using the R statistical software (4.0.3. version) [37], specifically the binom packages.

### 2.6. Phylogenetic Analysis

The maximum likelihood phylogenetic tree was created with the Tamura-3 substitution models with gamma-distributed sites, as suggested by the MEGAX software model test [35,36], based on 1000 bootstrap replications. The sequences obtained in this study were submitted to NCBI GenBank under the following accession numbers: from MW080960 to MW080970.

## 3. Results

### 3.1. TTSuV Prevalence in Wild Ungulates and Associated Risk Factors

A total of 528 wild ungulate liver samples were analyzed (*n* = 400 from wild boars, and *n* = 128 from wild ruminants), and the overall prevalence was calculated (Table 4).

Overall TTSuV prevalence was 5.7% (30/528, 95% CI 3.9–8.0%). A higher prevalence (*p*-value < 0.05) was found in wild ruminants (9.4%, 95% CI 4.9–15.8%) compared to wild boars (4.5%, 95% CI 2.7–7.0%). No risk factor was associated between TTSuV presence and either gender or age class of wild animals (*p*-value > 0.05). Considering sampling areas, a higher prevalence (*p*-value < 0.05) was found in wild ungulates in Sondrio Province (7.4%, 95% CI 4.9–10.6%) compared to the Parma area (2.3%, 95% CI 0.6–5.7%); TTSuV prevalence in wild boars (data not shown) was also higher in the mountains than in the flatland (14/225, 6.2%, 95% CI 3.7–10.2 in Sondrio Province, and 4/175, 2.3%, 95% CI 0.6–5.7% in the Parma area). Finally, the highest prevalence was observed in the 2016–2017 hunting season (13.9%, 95% CI 8.3–21.4%), followed by a decrease throughout the 2019–2020 hunting season (2.7%, 95% CI 0.7–6.7%), (*p*-value < 0.05).

### 3.2. TTSuV1a and TTSuVk2a Prevalence

TTSuV1a and TTSuVk2a prevalence was investigated in wild boars and wild ruminants according to gender, age class, hunting season, and sampling area.

The overall prevalence in wild ungulates was 2.1% (11/528, CI95% 1.2–3.7%) for TTSuV1a and 4.2% (22/528, CI95% 2.8–6.2%) for TTSuVk2a (data not shown). 

Results for TTSuV1a and TTSuVk2a detection in wild boars are shown in Table 5.

In wild boars, TTSuV1a was detected in 11/400 samples (2.7%, CI95% 1.4–4.9%), while TTSuVk2a was detected in 10/400 samples (2.5%, CI95% 1.2–4.5%). In three wild boar samples (0.75%, CI95% 0.3–2.2%) TTSuV1a and TTSuVk2a co-infection was observed; two samples were found in Sondrio Province and one in the Parma area (data not shown).

Considering wild boar gender, TTSuV1a and TTSuVk2a prevalence in males was 3.2% (CI95% 1.3–6.6%) and 3.7% (CI95% 1.6–7.2%), respectively. In females, TTSuV1a prevalence was 2.2% (CI95% 0.6–5.5%), while TTSuVk2a prevalence was 1.1% (CI95% 0.1–3.9%). No positive hepatic sample was found in pregnant animals. Moreover, TTSuV1a was detected in 1.7% (CI95% 0–8.9%) of class 0 animals and in 2.9% (CI95% 1.0–6.7%) of both class 1 and 2 animals. TTSuVk2a was detected with 5.0% prevalence (CI95% 1.0–13.9%) in class 0, 2.9% (CI95% 1.0–6.7%) in class 1, and 1.2% (CI95% 0.1–4.2%) in class 2.

In the 2018–2019 hunting season, a prevalence of 5.3% (CI95% 2.8–9.8%) and 4.1% (CI95% 2.0–8.3%) was observed for TTSuV1a and TTSuVk2a, respectively, while in the 2019––2020 hunting season prevalence decreased to 0.9% (CI95% 0.2–3.1%) for TTSuV1a, and 1.3% (CI95% 0.4–3.7%) for TTSuVk2a (Table 5).

In wild boars from Sondrio Province, both TTSuV1a and TTSuVk2a prevalence was 3.6% (CI95% 1.5-6.9%), while in the Parma area TTSuV1a was detected in 1.7% (CI95% 0.3–4.9%) samples, and TTSuVk2a in 1.1% (CI95% 0.1–4.1%) samples (Table 5).

In wild ruminants (red deer, roe deer, and chamois) from Sondrio Province, TTSuV1a was not detected, while TTSuVk2a prevalence was 9.4% (CI95% 4.9–15.8%) (Table 6).

TTSuVk2a showed a prevalence of 11.3% (CI95% 4.3–23.0%) in males and 8.0% (CI95% 3.0–16.6%) in females. TTSuVk2a prevalence in class 0 was 11.1% (Cl95% 3.1–26.1%); in class 1 it was 7.7% (Cl95% 0.9–25.1%), and in class 2 it was 9.1% (Cl95% 3.4–18.7%). From 2016–1017 to 2018–2019 the prevalence decreased from 13.8% (Cl95% 6.1–25.4%) to 2.6% (Cl95% 0.1–13.8%).

### 3.3. Phylogenetic Tree

**Figure 1:**
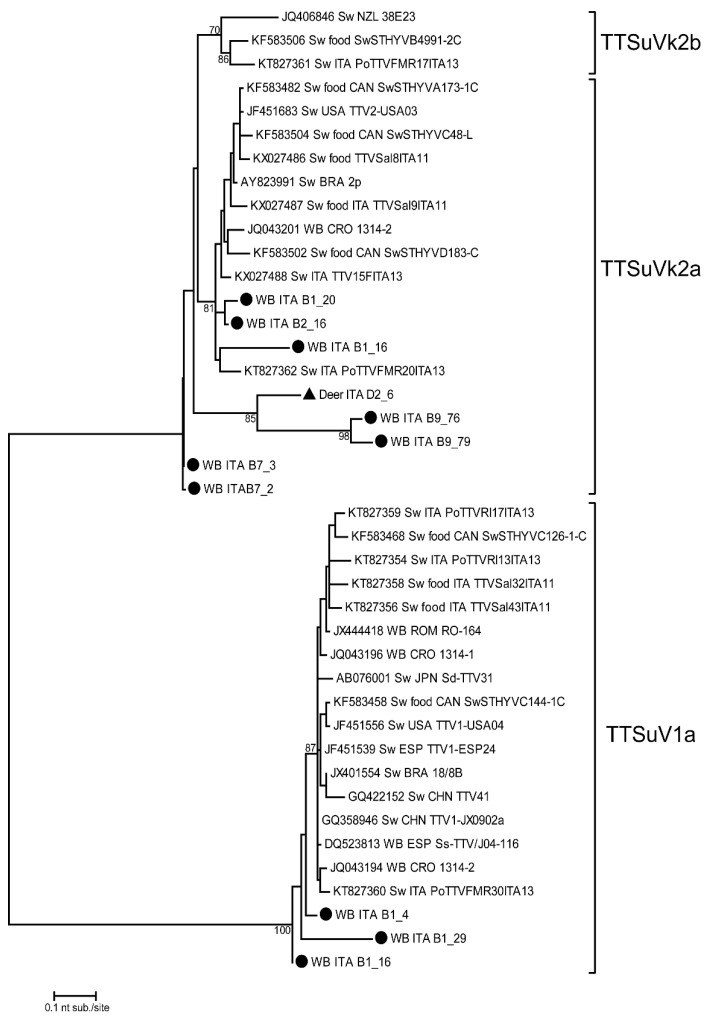
Phylogenetic tree of TTSuV 5′- end of the UTR (nt 138–453) nucleotide sequences. TTSuV strains of this study are indicated with a black circle for wild boars and a black triangle for red deer. Reference sequences are also considered in the dendrogram. The maximum likelihood phylogenetic tree was constructed with 1000 bootstrap repetitions. Bootstrap values under 70% are not shown. WB = wild boar; Sw = swine.

The phylogenetic analysis of TTSuV was performed on sequences obtained from 10 wild boars and 1 red deer liver sample, confirming the presence of both genogroups 1 and 2 in wild animals (Figure 1). In particular, TTSuV1a and TTSuVk2a were detected in 3 and 7 wild boar liver samples, respectively. In red deer, only one TTSuVk2a strain was detected. All sequences were obtained from animals infected with one viral genogroup, except for sample B1_16 (wild boar) with a TTSuV1a/TTSuVk2a co-infection.

The phylogenetic tree in Figure 1 reveals a high heterogeneity among the obtained nucleotide sequences. The wild boar strains belonging to TTSuV1a (B1_4, B1_16, and B1_29) had the same geographical and temporal origin (Sondrio Province in the 2018–2019 hunting season). These sequences shared an intra-group nucleotide identity (nt. id.) ranging between 86.2% and 96.7% and revealed the highest nt. id. with two TTSuV1a strains previously detected in Croatia (JQ043194) and Italy (KT827360) in wild boar and swine liver samples, respectively, and are related also to food products containing raw swine liver. The phylogenetic tree also shows a high nucleotide sequence heterogeneity when considering the TTSuVk2a clade. In fact, TTSuV strains detected in this study in wild boar and red deer were grouped in three distinct clades related to strains from several animal hosts. The first clade included three strains (B1_16, B1_20, and B2_16) detected in wild boars (clade nt. id. ranging between 88.3% and 97.5%) grouped with strains detected in domestic pigs in Italy and in wild boars in Croatia. The three strains from this study were all collected in Sondrio Province in the 2018–2019 hunting season. The second (B9_76, B9_79, and D2_6) and third clades (B7_2 and B7_3) included three (two from wild boars collected in the Parma area in 2019–2020, and one from red deer collected in Sondrio Province in 2017–2018) and two (from wild boars collected in the Parma area in 2019–2020) sequences showing between 75.6% and 84.5% nt. id. with other TTV reference strains.

## 4. Discussion

The aim of this study was to investigate the presence of TTSuV in wild ungulates in two areas in Northern Italy with different environmental and anthropic characteristics to better understand their role as reservoirs for the virus. Our investigation revealed the presence of TTSuV in wild fauna in both the selected areas. In the mountains (Sondrio area) our data show a prevalence of 7.4%, compared to a lower prevalence in the flatland (Parma area, prevalence of 2.3%). In the Sondrio area, low levels of biosecurity practices are performed in the sporadic farms, and pigs are also frequently bred in the pasture in the summer season, opening up the potential of possible interaction with wild fauna. The lower prevalence observed in wild ungulates in the Parma area could instead suggest a low risk of transmission from game animals to domestic pigs. Indeed, in this territory pigs are reared following high biosecurity levels, due to the presence of many factories involved in Parma ham production, one of the most important Italian excellences.

TTSuV prevalence was higher in wild ruminants (9.4%) than in wild boars (4.5%). Literature about TTSuV in wild fauna is lacking. To the best of our knowledge, this study shows the first data on TTSuV in wild ungulates in Northern Italy. According to our results, the only TTSuV detected in wild ruminants was TTSuVk2a (9.4%); nevertheless, due to the absence of data, further studies are needed to better define the prevalence of TTSuV in wild ruminants. In wild boars, both investigated genogroups were detected with a similar prevalence (2.7% for TTSuV1a, and 2.5% for TTSuVk2a) in the two analyzed areas. A comparable study performed in Romania reported higher prevalence (60% for TTSuV1a, and 32% for TTSuVk2a) in some wild boar tissues (also including liver) [27]. Although data on virus prevalence in hepatic tissue are lacking, some studies on sero-prevalence reported higher levels of antibodies against TTSuV1a and TTSuVk2a in wild boars in Europe. In particular, a sero-prevalence of 20% for TTSuV1a and 49% for TTSuVk2a was reported from wild boars in Germany, while higher values were found in Spain (57.8% for TTSuV1a and 66.3% for TTSuVk2a) [12,26].

The investigation on possible risk factors associated with TTSuV infection in wild ungulates showed no risk associated with animal gender. However, a higher prevalence trend in males (*p*-value > 0.05) was observed, despite paucity of data on TTSuV infection in wild animals. Surprisingly, no positive sample was found in pregnant females, thus suggesting that pregnancy could represent a protective factor against TTSuV infection or persistence. Moreover, no apparent correlation between TTSuV infection and age class was found, although an increasing infection rate with growing age is reported [25]. Finally, the decreasing trend observed in TTSuV infection rate in wild ungulates during the different hunting seasons (from 13.9% in 2016–2017 to 2.7% in 2019–2020) is difficult to explain. This trend may be related to the random sampling of wild animals belonging to isolated populations. In fact, the evolution and transmission dynamics of infections are difficult to define in isolate wild animal groups, unlike those of farmed animals in which a massive viral transmission is more likely. For all these reasons, further studies are necessary to better understand TTSuV transmission pathways in wild fauna.

In our study, TTSuV1a and TTSuVk2a co-infection was observed only in three wild boar liver samples (0.75%), but data from the literature are controversial. In fact, no co-infection of TTSuVs was observed in the lungs, livers, lymph nodes, and sera samples from wild boars in Uruguay, while a low co-infection (1.7%) was detected in domestic pig blood in Nigeria [38,39]. Other studies showed that TTSuV1a and TTSuVk2a co-infection was common in sera samples from wild boars, with a prevalence of 19% in Germany, 40% in Spain, and 24% in Romania, where samples of lung, liver, lymph node, kidney, and tonsil were also analyzed [12,26,27]. Finally, the livers of this study were tested in a previous study for *hepatits E virus* infection [40], but no co-infection was observed. However, since some recent studies assumed the capacity of TTSuV to enhance the severity of some diseases (i.e., caused by porcine circovirus type 2 or African swine fever virus [22,39], the potential pathogenic role of co-infection with TTSuV1a and TTSuVk2a and its possible role in the aggravation of the diseases should be further studied.

The sequencing of positive samples was successful only in a subset of the positive samples; this may possibly be due to a low initial viral titer, a complex matrix, or poor sample quality. To enhance the number of high-quality sequences viral isolation may be evaluated in the future prospects. However, a recent review reported the lack of a reliable in vitro culture system for TTSuV isolation both in human and pigs. In fact, just primary cultures of bone marrow and stimulated peripheral blood mononuclear cells could support the in vitro isolation and productive replication of huTTVs and TTSuVs, at the possible expense of genetic consistency [41]. The phylogenetic analysis confirmed the circulation of TTSuV strains in wild animals, with a strict correlation between strains detected in domestic pigs in Italy and in wild boars in Croatia, which was related to some food products containing raw swine liver. TTSuV1a sequences detected in our study were grouped and originated from wild boars living in the same province (Sondrio) and collected in the same hunting season (2018–2019). Focusing on TTSuVk2a, the first clade grouped wild boars from Sondrio Province collected in the same hunting season (2018–2019), while the other two clades grouped wild boars collected in 2019–2020 in the Parma area. All the TTSuVk2a sequences are related with swine strains, suggesting that the same strains circulate in these areas in the same years. Considering the different characteristics of the two sampling areas, the observed genetic correlation between strains previously identified in both wild and domestic pigs may suggest a possible direct or indirect viral transmission from domestic to wild animals or vice versa. This is particularly true for wild boars, as wild reservoirs belonging to the *Suidae* family, but other species may be involved, such as deer. Furthermore, considering the virus’s environmental stability, the sharing of the same habitat for food purposes by different species, including wild ungulates, could enhance environmental contamination, which in turn could lead to increased viral circulation [41,42]. Moreover, recent findings reported high levels of TTSuV and huTTV DNA in both humans and swine, suggesting that human exposure to TTSuVs could be due to the environment, water and pig feed contamination, proximity to swine, and pork product consumption [15,43,44].

## 5. Conclusions

TTSuV’s detection in the wild fauna of Northern Italy highlighted the need for monitoring studies to improve the knowledge of emerging viruses and to better evaluate their likely zoonotic potential. To assess the risk of transmission from domestic pigs to wild animals and vice versa, the number of species and animals tested should be increased. Future prospects should also include environmental and farmed animal samples to identify the main sources of contamination and better understand the virus circulation and distribution at the livestock–wildlife interface. Wild animals could be putative reservoirs that may contribute, to varying degrees, to the spread and amplification of viruses, possibly including those initially originated on farms, to other farms with eventual transmission to humans. Finally, focusing on analyses performed with full-length sequencing on a variety of samples (including environmental, wild, domestic, and human samples) would allow us to gain more insights about TTSuV evolution and epidemiology. The public health significance of TTSuVs as potentially zoonotic swine viruses must be evaluated, considering that modern human lifestyle, which is characterized by growing populations, high density rates, and global movements, leads to the increase in new risks for human and animal health.

## Figures and Tables

**Table 1:** Characteristics of the wild boars sampled in Sondrio (SO) and Parma (PR) areas divided by gender and age class. The number of pregnant females is reported in brackets.

Sampling Area	Gender	Hunting Season	Age Class ^1^			Total Samples
			0	1	2	
SO	Male	2018/2019	4	17	15	142
		2019/2020	22	57	27	
	Female	2018/2019	5	11	12	83
		2019/2020	8	30	17	
	Total samples		39	115	71	225
PR	Male	2018/2019	1	17	20	74
		2019/2020	13	6	17	
	Female	2018/2019	1	23 (6)	43 (32)	101 (38)
		2019/2020	6	9	19	
	Total samples		21	55	99	175

^1^ Class 0: young; class 1: sub-adult; class 2: adult.

**Table 2:** Characteristics of red deer, roe deer, and chamois sampled in Sondrio Province (SO), divided by gender and age class.

Gender	Hunting Season	Age Class ^1^			Total Samples
		0	1	2	
Male	2016/2017	7	10	4	53
	2017/2018	6	4	9	
	2018/2019	7	0	6	
Female	2016/2017	9	4	24	75
	2017/2018	3	4	6	
	2018/2019	4	4	17	
Total samples		36	26	66	128

^1^ Class 0: young; class 1: sub-adult; class 2: adult.

**Table 3:** Primers used for TTSuV1a and TTSuVk2a DNA amplification [34].

Target	Type	Sequence	Position	Amplicon Size (bp)
TTSuV1a	Forward	5′-CGGGTTCAGGAGGCTCAAT-3′	8–26	305
	Reverse	5′-GCCATTCGGAACTGCACTTACT-3′	291–312	
TTSuVk2a	Forward	5′-TCATGACAGGGTTCACCGGA-3′	1–20	252
	Reverse	5′-CGTCTGCGCACTTACTTATATACTCTA-3′	226–252	

**Table 4:** TTSuV prevalence and 95% confidence intervals (95% CI) are reported. Results are divided by species (wild ruminants include red deer, roe deer, and chamois), risk factors evaluated (animal gender and age class), sampling area, and hunting season.

Wild Ungulates (*n* = Samples)	TTSuV Prevalence (95% CI, *n* = Positive Samples)
Overall (*n* = 528)	5.7% (3.9–8.0%, *n* = 30)
Species	
Wild ruminants (*n* = 128)	9.4% (4.9–15.8%, *n* = 12)
Wild boars (*n* = 400)	4.5% (2.7–7.0%, *n* = 18)
Gender	
Female (*n* = 259)	4.2% (2.1–7.5%, *n* = 11)
Male (*n* = 269)	7.1% (4.3–10.8%, *n* = 19)
Age class ^1^	
0 (*n* = 96)	8.3% (3.7–15.8%, *n* = 8)
1 (*n* = 196)	5.1% (2.5–9.2%, *n* = 10)
2 (*n* = 236)	5.1% (2.6–8.7%, *n* = 12)
Sampling area	
Parma (*n* = 175)	2.3% (0.6–5.7%, *n* = 4)
Sondrio (*n* = 353)	7.4% (4.9–10.6%, *n* = 26)
Hunting season	
2016–2017 (*n* = 122)	13.9% (8.3–21.4%, *n* = 17)
2017–2018 (*n* = 32)	9.4% (2.0–25.0%, *n* = 3)
2018–2019 (*n* = 225)	2.7% (1.0–5.7%, *n* = 6)
2019–2020 (*n* = 149)	2.7% (0.7–6.7%, *n* = 4)

^1^ Class 0: young; class 1: sub-adult; class 2: adult.

**Table 5:** TTSuV1a and TTSuVk2a prevalence and 95% confidence intervals (95% CI) according to gender, age class, hunting season, and sampling area.

Wild Boars (*n* = Samples)	TTSuV1a Prevalence (95%CI, *n* = Positive Samples)	TTSuVk2a Prevalence (95%CI, *n* = Positive Samples)
Overall (*n* = 400)	2.7% (1.4–4.9%, *n* = 11)	2.5% (1.2–4.5%, *n* = 10)
Gender		
Males (*n* = 216)	3.2% (1.3–6.6%, *n* = 7)	3.7% (1.6–7.2%, *n* = 8)
Females (*n* = 184)	2.2% (0.6–5.5%, *n* = 4)	1.1% (0.1–3.9%, *n* = 2)
Age class ^1^		
0 (*n* = 60)	1.7% (0–8.9%, *n* = 1)	5.0% (1.0–13.9%, *n* = 3)
1 (*n* = 170)	2.9% (1.0–6.7%, *n* = 5)	2.9% (1.0–6.7%, *n* = 5)
2 (*n* = 170)	2.9% (1.0–6.7%, *n* = 5)	1.2% (0.1–4.2%, *n* = 2)
Hunting season		
2018–2019 (*n* = 169)	5.3% (2.8–9.8%, *n* = 9)	4.1% (2.0–8.3%, *n* = 7)
2019–2020 (*n* = 231)	0.9% (0.2–3.1%, *n* = 2)	1.3% (0.4–3.7%, *n* = 3)
Sampling area		
Sondrio (*n* = 225)	3.6% (1.5–6.9%, *n* = 8)	3.6% (1. 5–6.9%, *n* = 8)
Parma (*n* = 175)	1.7% (0.3–4.9%, *n* = 3)	1.1% (0.1–4.1%, *n* = 2)

^1^ Class 0: young; class 1: sub-adult; class 2: adult.

**Table 6:** TTSuVk2a prevalence and 95% confidence intervals (95% CI) in wild ruminants according to gender, age class, and hunting season.

Wild Ruminants *(n* = Samples)	TTSuVk2a Prevalence (95% CI, *n* = Positive Samples)
Overall (*n* = 128)	9.4% (4.9–15.8%, *n* = 12)
Gender	
Males (*n* = 53)	11.3% (4.3–23.0%, *n* = 6)
Females (*n* = 75)	8.0% (3.0–16.6%, *n* = 6)
Age class ^1^	
0 (*n* = 36)	11.1% (3.1–26.1%, *n* = 4)
1 (*n* = 26)	7.7% (0.9–25.1%, *n* = 2)
2 (*n* = 66)	9.1% (3.4–18.7%, *n* = 6)
Hunting season	
2016–2017 (*n* = 58)	13.8% (6.1–25.4%, *n* = 8)
2017–2018 (*n* = 32)	9.4% (2.0–25.0%, *n* = 3)
2018–2019 (*n* = 38)	2.6% (0.1–13.8%, *n* = 1)

^1^ Class 0: young; class 1: sub-adult; class 2: adult.

## Data Availability

The data presented in this study are available within the study itself.
